# Correlation of the Nutritional Risk Screening 2002 Score With Post-operative Complications in Gastrointestinal and Hepatopancreatobiliary Oncosurgeries

**DOI:** 10.7759/cureus.58514

**Published:** 2024-04-18

**Authors:** Yugal Limbu, Sneha Raut, Prashanta Pudasaini, Sujan Regmee, Roshan Ghimire, Dhiresh Kumar Maharjan, Prabin Bikram Thapa

**Affiliations:** 1 Department of Gastrointestinal and General Surgery, Kathmandu Medical College and Teaching Hospital, Kathmandu, NPL

**Keywords:** gastrointestinal, hepatopancreatobiliary, nutritional status, oncosurgery, post-operative complications

## Abstract

Introduction

The Nutritional Risk Screening 2002 (NRS 2002) is a reliable tool for assessing patients' nutritional status and for identifying those who may benefit from nutritional support before undergoing surgery. However, its application and correlation with post-operative outcomes for Nepalese patients undergoing gastrointestinal and hepatopancreatobiliary oncosurgeries remain unexplored. The objective of this study was to correlate the NRS 2002's nutritional risk with post-operative complications classified by the Clavien-Dindo Classification.

Methods

A prospective analytical study was conducted at Kathmandu Medical College and Teaching Hospital, with 74 adults who underwent gastrointestinal and hepatopancreatobiliary oncosurgeries between 1st March 2021 and 30th August 2022. The study was conducted following ethical clearance from the Institutional Review Committee of the Hospital. A convenience sampling method was used. Data were analyzed using IBM SPSS Statistics for Windows, Version 20 (Released 2011; IBM Corp., Armonk, New York, United States).

Results

Among the 122 patients admitted during the study period, 74 met the inclusion criteria. Using the NRS-2002, 37.8% were found to be at nutritional risk. Such patients had a higher risk of complications and extended hospital stays, supported by an odds ratio of 1.647 (95% confidence interval: 1.223 -2.219) and a p-value of <0.001. Nutritional risk emerged as an independent predictor of post-operative complications.

Conclusion

The study suggests the potential of NRS-2002 as a significant predictor of outcomes after surgeries for gastrointestinal and hepatopancreatobiliary malignancies in the South Asian context, particularly in Nepal. Tools such as NRS 2002 play a pivotal role in early risk identification, which could subsequently influence both pre-operative and post-operative care strategies, ultimately enhancing patient outcomes.

## Introduction

Nutritional support for surgical patients requires understanding metabolic changes in the post-operative period, as poor nutrition is linked to post-operative complications [1]. Starvation from injury differs from physiological fasting, with surgery causing an inflammatory and metabolic stress response [2]. Such responses release stress hormones and inflammatory mediators, notably cytokines, that instigate the systemic inflammatory response syndrome [2]. This affects metabolism, causing the breakdown of glycogen, fat, and protein, resulting in muscle tissue loss and affecting short- and long-term recovery [2,3]. Nutrition is vital for cancer patients, especially post-surgery.

Malnutrition in these patients is common and relates to increased post-operative complications, extended hospital stays, and reduced survival rates [4]. Several tools assess nutritional status, with the Nutritional Risk Screening 2002 (NRS 2002) recommended for cancer and surgical patients [1].

This study investigates the link between the NRS 2002 score and post-operative complications in the South Asian population, with a particular focus on the Nepalese population undergoing gastrointestinal (GI) and hepatopancreatobiliary oncosurgeries.

## Materials and methods

Study design and population

This was a prospective analytical study, conducted on 122 patients who received surgical treatment for GI and hepatopancreatobiliary malignancies at Kathmandu Medical College and Teaching Hospital (KMCTH), Sinamangal, Kathmandu, Nepal from 1st March 2021 to 30th August 2022. Ethical clearance was obtained from the Institutional Review Committee of KMCTH (Reference number: 3112202002). 

Inclusion criteria 

All patients aged 18 years and above who underwent curative surgery for GI and hepatopancreatobiliary malignancies were included in the study after obtaining written informed consent. 

Exclusion criteria

Patients with an irresectable tumor at the time of operation, uncontrolled diabetes mellitus at admission (HbA1C >9 gm/dl), American Society of Anesthesiologists (ASA) grade IV and V patients, those who had undergone neoadjuvant chemotherapy and/or radiotherapy, or were on steroid therapy, and those who did not consent to the study were excluded. 

Sample size determination

**Figure 1:**
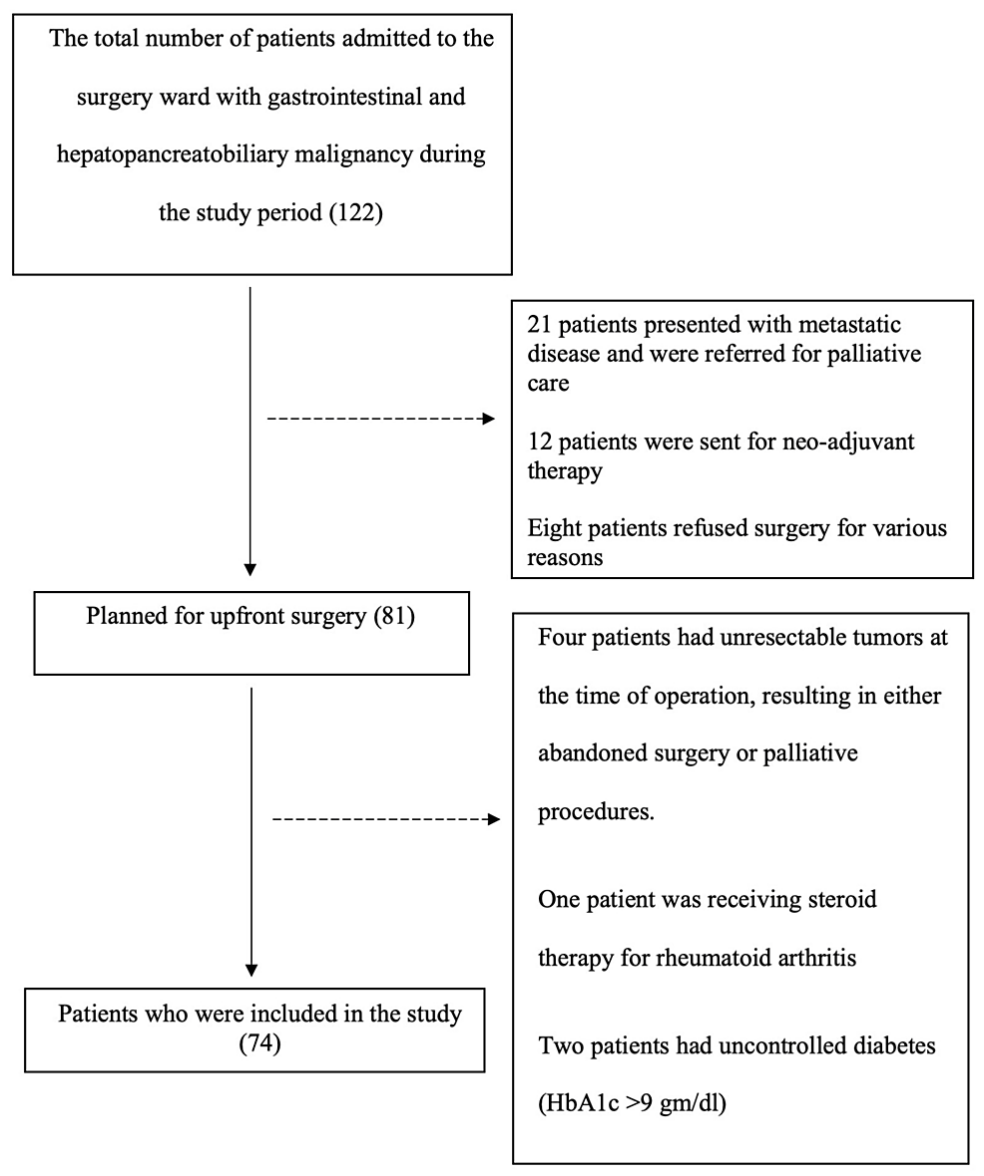
Patient recruitment process HbA1c: Hemoglobin A1c

Cochran’s formula n=(Z^2^pq)/e^2^ was utilized for sample size calculation, where Z represents the standard normal deviation for a desired confidence level (in this case, 1.96 for a 95% confidence interval), p denotes the prevalence of post-operative complication as obtained from the referenced study (0.26) [5], q is the complementary probability of p (1-p, which equals 0.74), and e signifies the margin of error (10%). Substituting the respective values into the formula, the calculated sample size was determined to be 74. A convenience sampling method was used. The patient recruitment process is demonstrated in Figure 1. 

Data collection

A comprehensive medical history was recorded, followed by a detailed clinical examination upon the patient's admission to the surgical ward. All relevant pre-operative investigations were conducted. The patient's body height was measured to the nearest 0.5 cm with a calibrated stadiometer in the ward, while weight was measured to the nearest 0.5 kg with a calibrated weighing scale. The body mass index (BMI) was then calculated based on the recorded height and weight measurements. On the day preceding the scheduled surgery, the NRS 2002 proforma was used to classify patients who were nutritionally "at risk". Patients with age-corrected total scores of three or more in NRS screening are considered to be at nutritional risk.

Post-operative assessment

Post-operatively, the incidence of complications in the patient until discharge was documented using the Clavien-Dindo classification system. The Clavien-Dindo classification system categorizes surgical complications by severity, offering a standardized method for grading adverse events. Grades range from I to V, with each grade indicating the severity of the complication [6]. A higher complication grade was recorded if the patient had more than one complication. The patient's particulars, the operative procedure performed, the final histopathological diagnosis, and other relevant information were also recorded. The NRS 2002 risk category was then correlated with the grade of post-operative complication, and the statistical significance between the two variables was analyzed.

Statistical analysis 

The obtained data were entered and analyzed using IBM SPSS Statistics for Windows, Version 20 (Released 2011; IBM Corp., Armonk, New York, United States). Categorical data were analyzed using the chi-square test, while continuous data were analyzed using the t-test. Measures of central tendency, including mean, median, and mode, were calculated as appropriate. For regression assessment, bivariate and multivariate logistic regression analyses were used. The correlation was assessed through chi-square analysis. A p-value of less than 0.05 was considered significant for all statistical analyses.

## Results

Patient characteristics

The study comprised a cohort of 122 patients with GI and hepatopancreatobiliary malignancies. Of these, 74 consecutive patients who met the inclusion criteria and underwent curative resection were included in the analysis.

Prevalence of nutritional risk and complications

The prevalence of patients at nutritional risk, assessed by the NRS-2002 screening tool, was 37.8% (28 out of 74 patients), out of which two (2.7%) mortalities and nine (12.2%) major complications, not including death (Clavien-Dindo grade 3 and 4) were recorded. Table 1 displays a comparison of patients’ complication grades and average hospital stay lengths based on their nutritional risk levels.

**Table 1:** Comparison of patients at nutritional risk and low risk with regard to the grade of complications and average length of hospital stay *Refers to a significant result

	Clavien-Dindo Grade of Complication						Average Length of Stay	p-value
	0	1	2	3	4	5		
Nutritionally at low risk	26	5	15	0	0	0	6.8 days	
Nutritionally at risk	9	0	8	8	1	2	11.9 days	<0.001*
Total	35	5	23	8	1	2		

The most common major complication which required intervention was pleural effusion. The mean BMI of the patients was 22.3kg/m^2^ and their mean serum albumin level was 3.7 g/dL. Baseline characteristics of the patients are shown in Table 2. 

**Table 2:** Baseline characteristics of the patients ECOG: Eastern Cooperative Oncology Group; ASA: American Society of Anesthesiologists

Performance Status (ECOG)	Frequency	Percent
0	2	2.7
1	60	81.1
2	12	16.2
Total	74	100
ASA	Frequency	Percent
1	38	51.4
2	36	48.6
Total	74	100
Tumor Staging	Frequency	Percent
I	16	21.6
II	21	28.4
III	24	32.4
IV	13	17.6
Total	74	100
Resection Margin	Frequency	Percent
R0	71	95.9
R1	3	4.1
Total	74	100
Clavien Dindo Classification	Frequency	Percent
0	35	47.3
1	5	6.8
2	23	31.1
3a	7	9.5
3b	1	1.4
4	1	1.4
5	2	2.7
Total	74	100

Malignancy profile and association of nutritional risk with outcomes

The most common malignancy requiring curative resection was periampullary tumors (47.2%), followed by colorectal (20.3%), gastric (13.6%), gallbladder and proximal bile duct (13.5%), liver (4%), and esophageal (1.4%). Patients at nutritional risk exhibited a statistically significant increased risk of morbidity and mortality, as evidenced by an odds ratio (OR) of 1.647 (95% confidence interval: 1.223 -2.219) and a p-value of <0.001. There were two in-hospital mortalities among the 74 patients included in the analysis, resulting in a mortality rate of 2.7%. Additionally, the nutritional risk was identified as an independent predictor of post-operative complications and increased length of hospital stay in this population.

In bivariate analysis, patients at nutritional risk as per NRS-2002 had a significant association with major complications in the post-operative period (p-value < 0.001). Among the considered confounding variables, serum albumin, ASA grade, and ECOG performance status demonstrated a statistically significant association with patients developing major post-operative complications. In contrast, the patient's age, BMI, tumor stage, and resection margin did not show significant associations. However, in multivariate analysis, only an NRS score equal to or greater than three, and low serum albumin (<3.5) had a statistically significant correlation with major post-operative complications (OR- 1.647 {95% confidence interval: 1.223 -2.219}) (Tables 3, 4).

**Table 3:** Correlation of major complications and variables having non-Gaussian distribution *Refers to a significant result

	Major Complications						p-value
	Yes			No			
	Median	Q1	Q3	Median	Q1	Q3	
Age	67	56	72	60	54	67	0.334
Height (cm)	162	160	172	160	155	164	0.152
Weight (kg)	55	52	62	57	50	62	0.879
BMI (kg/m^2^)	20.8	19.2	21.5	22	20.3	24.2	0.055
Serum Albumin (g/dl)	3	2.7	3.1	3.9	3.6	4.1	<0.001*

**Table 4:** Correlation of major complications and variables having Gaussian distribution ASA: American Society of Anesthesiologists *Refers to a significant result

	Major Complications				p-value
	Yes	%	No	%	
Sex					0.21
Female	3	27.3	30	47.6	
Male	8	72.7	33	52.4	
Impaired nutritional status score					
Absent	0	0.0	27	42.9	<0.001*
Mild	1	9.1	24	38.1	
Moderate	8	72.7	10	15.9	
Severe	2	18.2	2	3.2	
Nutritional category					
Low risk	0	0.0	46	73.0	<0.001*
At risk	11	100.0	17	27.0	
Tumor Staging					
I	2	18.2	14	22.2	0.421
II	2	18.2	19	30.2	
III	3	27.3	21	33.3	
IV	4	36.4	9	14.3	
Resection Margin					
R0	10	90.9	61	96.8	0.929
R1	1	9.1	2	3.2	
Performance status					
0	0	0.0	2	3.2	0.002*
1	5	45.5	55	87.3	
2	6	54.5	6	9.5	
ASA grade					
1	0	0.0	38	60.3	<0.001*
2	11	100.0	25	39.7	

## Discussion

In this prospective study involving 74 patients undergoing surgical procedures for hepatopancreatobiliary or gastrointestinal cancer, it was noted that 14.9% experienced major complications, while 85.1% had minor ones. This observed complication rate aligns with the results from other renowned American and European cancer centers [7,8]. Notably, 37.8% of these patients were identified as having potential nutritional risks according to the NRS 2002 screening tool. This risk correlated directly with the severity of complications. Patients with higher NRS scores experienced significantly more severe post-surgical challenges. This conclusion mirrors previous research that highlights a strong link between nutritional risk and the severity of post-operative complications [9]. Supporting this observation, another study reported the NRS-2002's accuracy in predicting post-surgery complications [10]. Their research additionally showcased a 40% prevalence of nutritional risk among cancer patients [10]. A crucial ripple effect of such risks was a prolonged hospital stay; a sentiment echoed in several other studies [4,9]. In a comprehensive analysis, when accounting for factors like ECOG and ASA grade and serum albumin levels, the NRS-2002 score still emerged as a critical predictor of post-surgical complications [11]. Particularly, patients with low serum albumin levels had heightened complication rates, emphasizing the importance of enhanced nutritional care for this cohort.

The landscape of research is replete with studies corroborating the above findings. For instance, several studies using different nutritional assessment tools consistently underline a direct relationship between compromised nutritional health and poor post-surgical outcomes [12,13]. In the realm of gastric cancer, providing targeted nutritional support significantly lowered infection rates and reduced hospital stays [14]. Moreover, early initiation of oral nutrition has been linked with better patient outcomes, and specific nutrition strategies, like immune-nutrition formulas rich in omega-3 polyunsaturated fatty acids, have demonstrated reduced post-operative complications [15].

For patients with esophageal tumors, weight loss and muscle mass depletion have been tied to unfavorable treatment results [4]. Initiating oral nutrition early has been advocated for these patients, with certain studies hinting at the possible benefits of specific supplements, like vitamin D, to mitigate post-surgical challenges [16]. Furthermore, in colorectal cancer scenarios, initiating nutritional treatment early post-surgery has been identified as a crucial survival predictor [4]. The positive impact of preoperative nutritional optimization, especially using specific formulations, has also been illuminated in various studies [4,17].

The NRS 2002 tool, endorsed by the European Society for Clinical Nutrition and Metabolism (ESPEN), is particularly notable [1]. It combines simplicity with effectiveness. Developed based on evidence-backed guidelines, the NRS 2002 tool provides a comprehensive insight into a patient's nutritional status, considering age, weight loss, and the presence of chronic diseases [18]. Its adoption by the ESPEN is a testament to its efficacy in spotting patients at potential malnutrition risk.

In addition to the NRS 2002 tool, other tools for nutritional assessment in clinical settings include the Subjective Global Assessment (SGA), Mini Nutritional Assessment (MNA), and Malnutrition Universal Screening Tool (MUST) [19]. While the NRS 2002 focuses specifically on nutritional risk screening, the Comprehensive Geriatric Assessment (CGA) is designed to evaluate a broader range of issues, such as medical comorbidities, functional status, and psychosocial capacities [19]. The relationship between NRS 2002 and CGA lies in their shared goal of identifying and addressing the complex needs of older adults, particularly those who are frail or at risk of adverse health outcomes [19,20].

However, while our study offers significant insights, it is essential to consider its limitations. Drawn from a single institution and relying on a limited sample size, the study might be influenced by potential biases and confounding variables. Factors like patients' age, cancer stage, comorbidity, and performance could affect the outcomes. Moreover, reliance on self-reported data for weight loss and calorie intake might introduce recall bias. Yet, objective measurements like BMI during admission validate our findings.

## Conclusions

This study highlights the crucial significance of nutritional assessment in the context of surgical outcomes for cancer patients, particularly those undergoing gastrointestinal and hepatopancreatobiliary oncosurgeries. By correlating the NRS 2002 score with post-operative complications, we underscore the importance of early risk identification for optimizing patient care strategies. The findings emphasize the potential of NRS 2002 as a valuable predictor of outcomes in the South Asian setting. Moving forward, integrating comprehensive nutritional assessment tools into routine clinical practice could substantially enhance the management and outcomes of surgical patients, offering a pathway toward improved care standards in oncological surgery.
